# Tenofovir and Doravirine Are Potential Reverse-Transcriptase Analogs in Combination with the New Reverse-Transcriptase Translocation Inhibitor (Islatravir) Among Treatment-Experienced Patients in Cameroon: Designing Future Treatment Strategies for Low- and Middle-Income Countries

**DOI:** 10.3390/v17010069

**Published:** 2025-01-06

**Authors:** Alex Durand Nka, Yagai Bouba, Wilfried Rooker Tsapi Lontsi, Davy-Hyacinte Gouissi Anguechia, Georges Teto, Aude christelle Ka’e, Ezechiel Ngoufack Jagni Semengue, Collins Ambe Chenwi, Désiré Takou, Lum Forgwei, Tatiana Anim-Keng Tekoh, Aurelie Minelle Kengni Ngueko, Bernadette Bomgning Fokou, Jeremiah Efakika Gabisa, Michel Carlos Tommo Tchouaket, Willy Leroi TognaPabo, Derrick Tambe Ayuk Ngwese, Jacky Njiki Bikoi, Daniele Armenia, Vittorio Colizzi, Marcel Yotebieng, Nicaise Ndembi, Maria-Mercedes Santoro, Francesca Ceccherini-Silberstein, Carlo-Federico Perno, Alexis Ndjolo, Joseph Fokam

**Affiliations:** 1 Chantal BIYA International Reference Centre for Research on HIV/AIDS Prevention and Management, Yaoundé P.O. Box 3077, Cameroon; romeobouba@yahoo.fr (Y.B.); tsapi.wilfred@gmail.com (W.R.T.L.); davygouissi@gmail.com (D.-H.G.A.); ggteto@yahoo.fr (G.T.); kae.audechristelle@gmail.com (A.c.K.); ezechiel.semengue@gmail.com (E.N.J.S.); collinschen@yahoo.co.uk (C.A.C.); dtakou@yahoo.com (D.T.); lforgwei1@gmail.com (L.F.); tatianatekoh@gmail.com (T.A.-K.T.); aurelieminel423@gmail.com (A.M.K.N.); efakikagabisa@gmail.com (J.E.G.); tommomichel@yahoo.fr (M.C.T.T.); willypabo@yahoo.fr (W.L.T.); derricktabeayuk@gmail.com (D.T.A.N.); colizzi@bio.uniroma2.it (V.C.); cf.perno@uniroma2.it (C.-F.P.); andjolo@yahoo.fr (A.N.); 2 Faculty of Medicine, UniCamillus-Saint Camillus International University of Health Sciences, 00131 Rome, Italy; daniele.armenia@gmail.com; 3 Department of Microbiology, Faculty of Science, University of Yaoundé 1, Yaoundé P.O. Box 337, Cameroon; j.njikibikoi@yahoo.fr; 4 Department of Experimental Medicine, University of Rome Tor Vergata, 00133 Rome, Italy; santormaria@gmail.com (M.-M.S.); ceccherini@med.uniroma2.it (F.C.-S.); 5 Department of Immunology, Molecular Medicine and Applied Biotechnology, University of Rome Tor Vergata, 00133 Rome, Italy; fokounathalie2@gmail.com; 6 Department of Medicine, Albert Einstein College of Medicine, Bronx, NY 10461, USA; marcel.yotebieng@einsteinmed.edu; 7 Africa Centres for Disease Control and Prevention, Addis Ababa P.O. Box 3243, Ethiopia; nicaisen@africa-union.org; 8 Microbiology and Diagnostic Immunology Unit, Bambino Gesù Children’s Hospital, IRCCS, 00165 Rome, Italy; 9 Central Technical Group, National AIDS Control Committee, Yaoundé P.O. Box 1459, Cameroon

**Keywords:** HIV, Islatravir, treatment-experienced patients, Cameroon

## Abstract

Islatravir (ISL) is a novel antiretroviral that inhibits HIV-1 reverse transcriptase translocation. The M184V mutation, known to reduce ISL’s viral susceptibility in vitro, could arise from prolonged exposure to nucleoside reverse transcriptase inhibitors (NRTI) (3TC). This study evaluated the predictive efficacy of ISL and identified potentially active antiretrovirals in combination among treatment-experienced patients in Cameroon, where NRTIs (3TC) have been the backbone of ART for decades now. Although ISL is a long-acting antiretroviral, it will provide other therapeutic options in combination with other reverse transcriptase inhibitors that remain effective. We analyzed 1170 HIV-1 sequences from patients failing first-, second-, and third-line ART using the CIRCB Antiviral Resistance Evaluation (CIRCB-CARE) database. Drug resistance mutations (DRMs) were interpreted using Stanford HIVdb.v9, and covariation patterns between M184V and major NRTI/NNRTI DRMs were assessed. The study population, with a median age of 40 years, showed a high prevalence of resistance to NRTIs (77.4%) and NNRTIs (49.2%). The most frequent NRTI DRMs were M184V/I (83.3%), M41L (25.0%), and T215FY (36.8%), while common NNRTI DRMs included K103NS (53.3%), Y181CIV (27.7%), and G190ASE (22.2%). In first-line ART failure, M184V significantly covaried with K70R, L74I, and M41L for NRTIs and K103N and G190A for NNRTIs. In second-line failure, the covariation with M184V extended to T215Y, M41L, and D67N for NRTIs and G190A, K103N, and K103S for NNRTIs. No significant covariation with M184V was observed in third-line treatment failures. Based on these covariations and on the effect of these mutations on available anti-HIV drugs, TDF (partial efficacy) and Doravirine (fully active) were identified as potentially suitable candidates in combination with ISL among patients failing the first, second, and third lines, and could serve as a valuable therapeutic option in LMICs facing similar treatment challenges.

## 1. Introduction

Improvements in antiretroviral therapy (ART) have played a crucial role in decreasing morbidity, mortality, and the transmission of HIV-1. However, HIV continues to pose a significant threat, particularly in Western and Central Africa, where an estimated 130,000 (100,000–170,000) out of the 630,000 (500,000–820,000) worldwide AIDS-related deaths occurred in 2023 despite increased access to antiretroviral therapy (ART) [1]. This region remains disproportionately affected by the pandemic, carrying over 12.7% (5.1 million people living with HIV) of the burden. Current efforts to achieve viral suppression, a crucial component of the “Test and Treat” strategy in resource-limited settings, face challenges [2]. While ART has expanded, viral suppression rates remain below the desired 95% target, currently at 89.2% for adults [2].

Factors contributing to this suboptimal rate include treatment non-adherence [3], interruptions in therapy [4], and high initial viral load [5]. These factors increase the risk of developing drug-resistant viral strains, further complicating treatment efforts [6]. To achieve the global goal of HIV elimination by 2030, a continued focus on improving viral suppression rates is essential and, therefore, requires a comprehensive approach to address the underlying causes of poor therapeutic outcomes, such as enhancing adherence to treatment, mitigating treatment interruptions, and managing high baseline viral load [7]. Concerning resistance profiles according to ART lines in Cameroon, a laboratory-based study with 759 patients (≥15 years) experiencing virological failure was carried out in Yaoundé, Cameroon. The overall acquired drug resistance was high (93.4% first line; 92.9% second line). The prevalence of drug resistance by antiretroviral class was 85.8% for NRTI and 90.3% for NNRTI. The most prevalent mutation was M184V (75.5%), followed by K103N (47.4%) and Y181C (27.7%) [8]. This highlights the necessity to reinforce treatment adherence and strengthen the genetic barrier of newly conceived ARV drugs, as the recommended HIV-1 treatment and prevention strategies rely on good adherence to daily administration; thus, agents with more favorable and extended dosing schedules would be of benefit [7]. Novel agents with improved safety and tolerability profiles and a high barrier to the selection of resistance are needed, as existing agents can be associated with long-term toxicities, as well as the potential for the selection of drug-resistant HIV-1 variants. Long-acting antiretroviral (LAAR) drugs are capable of being administered on a monthly or even less frequent basis, giving them the potential to improve adherence to therapy and extend opportunities for therapeutic or prophylactic intervention to underserved patient populations [7,9].

Islatravir (ISL) is the first nucleoside reverse transcriptase translocation inhibitor (NRTTI), with structural and mechanistic features that distinguish it from the currently marketed antiretroviral drugs. Its action is rooted in three structured chemical components (3′-OH group, 4′-ethynyl group, and 2-fluoro group) that contribute to its unique profile [10,11,12]. The OH group inhibits Islatravir metabolism and contributes to its long half-life, giving a 56 mg injection a half-life of 198 h and an ISL-eluting implant projected to release adequate ISL for more than 52 weeks [13]. Islatravir, considering these capacities, is a promising approach, especially for resource-limited settings (RLS) like Cameroon where ART is built on NRTIs (2NRTIs + NNRTI for the first line, 2NRTIs + PI for the second line, and 2NRTIs + INSTI for the third line) and, consequently, where resistance to this group of drugs is becoming alarming. Although it is a long-acting antiretroviral, it will provide other therapeutic options in combination with other reverse transcriptase inhibitors that remain effective. Recent clinical trials evaluating the investigational combination of Islatravir, as well as Lenacapavir, a first-in-class HIV-1 capsid inhibitor, have shown that a once-weekly oral combination regimen of Islatravir and Lenacapavir maintained viral suppression in adults at week 48 [14]. However, despite the strong genetic barrier of ISL compared to other reverse transcriptase inhibitors, some viral resistance selection studies [15,16] showed that Islatravir is not immune to resistance. These studies identified some mutations and combinations of mutations within RT that were shown to alter susceptibility to ISL in vitro. M184V and M184I conferred the largest fold reductions in potency to ISL (fold-change (FC) of 6.2 and 6.8, respectively) compared to the wild type, and only combinations of variants containing M184I or M184V conferred reduced potencies greater than 6.8-fold [16]. The effect of M184V/I highlights the importance of the cross-resistance within the class of NRTIs and Islatravir (NNRTI), which increases the risk of resistance to ISL among ISL-naive patients with previous exposure to NRTI. HIV drug resistance (HIVDR) is prevalent in Cameroon in drug-failing patients, with 89.1% of patients failing first-line ART exhibiting HIVDR, with 83.2% harboring the M184V mutation [17]. Pre-treatment drug resistance (PDR) is also common, with a 15.0% rate, primarily driven by NNRTI PDR (12.4%) [8].

Of note, the M184V substitution changes the geometry of the YMDD motif, which is located at positions 183 to 186 in RT. This motif is, in general, conserved among all retroviruses. Regarding other medications, a study of 255 clinical isolates with M184V revealed that this mutation did not lead to widespread resistance to NRTIs other than 3TC [18]. Nonetheless, M184V imparts low-level resistance to abacavir (ABC), ddI, and ddC according to tissue culture results [19,20,21,22]. In order to attain higher levels of resistance to the aforementioned drugs, other substitutions in RT are required, e.g., K65R, L74V, Y115F, and Q151M for resistance to ABC [21,23]. Higher-level resistance to ddI and ddC is often associated with M184V, L74V, and K65R substitutions in tissue culture. The presence of M184V during highly active antiretroviral therapy (HAART) with various combinations of drugs or in vitro during drug resistance selection experiments in cell cultures is associated with the reversal of resistance to certain drugs, e.g., Zidovudine (AZT), Stavudine (d4T), and tenofovir (TDF). Moreover, M184V is known to have antagonistic and suppressor effects for a variety of NRTI mutations, particularly those that are responsible for AZT resistance [24,25].

Therefore, regarding the long exposure and the high-level resistance to NRTIs (particularly 3TC, FTC, and, to a lesser extent, ABC) in Cameroon, a resource-limited setting where clinical trials are not yet performed for this new drug, and in the absence of universal access to resistance testing (a problem shared with the entirety of sub-Saharan Africa), there is the need to evaluate and monitor the co-occurrence of M184V and other major reverse transcriptase mutations, providing preliminary data on the efficacy of ISL and best potential treatment combinations among HIV-treated patients failing ART in Cameroon. Here, we report the results of a cross-sectional analytical study designed to assess the potential level of efficacy of ISL and the best potentially active RTIs in combination with ISL in Cameroon and similar resource-limited settings.

## 2. Materials and Methods

### 2.1. Specimen Used for Analysis

This study analyzed 1170 patient’s HIV-1 reverse transcriptase (RT) sequences obtained from the Chantal BIYA International Reference Center for research on the HIV/AIDS prevention and management (CIRCB) Antiviral Resistance Evaluation (CIRCB-CARE) database in Cameroon. These sequences were collected from patients failing first-line (2NRTI + 1NNRTI, *n* = 671), second-line (2NRTI + 1PI/r, *n* = 470), and third-line ART (2NRTI + 1INSTI = 29) between 2016 and 2023. Patients were either on first-, second-, or third-line ART. Only patients with a clearly documented treatment history (available in their medical record) were enrolled; patients with a negative genotypic resistance test (non-amplified samples) were excluded from this study, as well as patients with HIV sequences that could not be interpreted; all participants undergoing treatment were experiencing virological failure (i.e., a sustained plasma viral load > 1000 copies/mL).

### 2.2. Subtyping and Drug Resistance Determination

Nucleotide sequences were aligned with subtype/CRF reference sequences from the Los Alamos National Laboratory (LANL) database using the CLUSTAL.W integrated into Bioedit version 7.2.5 software (https://bioedit.software.informer.com/7.2/, accessed on 8 February 2024). Following a comparison of each sequence to the subtypes and CRF reference sequences (database accessed on 8 February 2024), gaps were removed from the final alignments. The phylogenic tree was constructed using Nextstrain–Nextclade version 3.10.0 Software [26] (https://clades.nextstrain.org, accessed on 8 February 2024).

### 2.3. Mutation’s Prevalence

HIV-1 sequences were analyzed using SeqScape (SeqScape software V3.0 initial license), and DRMs were interpreted using the Stanford HIVdb.v9 (https://hivdb.stanford.edu/hivdbby-mutations/, accessed on 12 February 2024) to determine the prevalence of M184V/I and other major RTIs-DRMs.

### 2.4. Mutation’s Covariation

Using R 4.4.1 software, pairwise interactions between the M184V mutation and other DRMs associated with NRTIs and NNRTIs were analyzed. Fisher’s exact test was employed to determine if the co-occurrence of these mutated residues differed significantly from what would be expected if they were independent events. Furthermore, the binomial correlation coefficient (phi) was calculated to quantify the strength of the correlation between M184V and other major RT mutations [27]. The Benjamini–Hochberg method was applied once more to adjust for multiple testing, utilizing a false discovery rate of 0.05. Samples containing a combination of two or more mutations at specific position pairs were excluded from the covariation calculations, as it is not feasible to determine whether these mutations are present in the same viral genome.

### 2.5. Deduction of Potential ARV in Combination

We utilized the Stanford HIVdb.v9 of major drug resistance mutations for NRTIs and NNRTIs to assess the susceptibility profiles of our study population to each antiretroviral (ARV). This assessment considered major mutations that exhibited significant correlations with M184V. For each drug, the impact of a mutation was categorized as follows: high-level reduced susceptibility, reduced susceptibility, reduced susceptibility in combination with other NRTI-resistant mutations, increased susceptibility, or no effect.

### 2.6. Cluster Analysis

To visualize the covariation patterns of mutations in greater detail, we employed average linkage hierarchical agglomerative clustering, a technique commonly used in phylogenetic tree construction [28]. This method constructs clusters of increasing size by iteratively merging pairs of clusters based on minimum average inter-cluster distances. The distance between mutation pairs was derived from the phi correlation coefficient, where a value of 1 indicates a strong positive association and −1 indicates a strong negative association. This measure was transformed into a distance metric, with linear interpolation between these extremes [28]. To assess the robustness of the resulting dendrogram, a bootstrap analysis was performed by repeating the clustering process 100 times on randomly sampled subsets of the original sequence data. This analysis provides confidence values for each sub-tree within the dendrogram, indicating the stability of the observed clustering patterns [28]. For example, a bootstrap value of 1 indicates that the two mutations (or groups of mutations) were consistently grouped together across all 100 bootstrap replications.

### 2.7. Ethical Approval and Informed Consent

This study was conducted in accordance with the Declaration of Helsinki. The study protocol was approved by the Cameroon Regional Ethics Committee of the Center region (ethical clearance CE N^o^ 0946/CRERSHC/2024). All biological data used in this study were extracted from the CIRCB-CARE database. Before accessing this database, we obtained authorization from the CIRCB research center to access and use these data, given that each patient consented to the use of their samples and data for future research during the different research works that required the samples and information.

## 3. Results

### 3.1. Demographic and Clinical Characteristics of Study Participants

Our study population consisted of 1170 PLHIV on ART, of whom 671 were on the first line, 470 on the second line, and 29 on the third line. Females were predominant (61.0%, 714/1170), and the overall median (IQR) age was 40 (28–47) years. The third line showed a lower age (17 (9–38) years) compared to those in the first (41 (33–47) years) and second lines (39 (20–38) years). The overall median (IQR) CD4 cell count was 190 (63–665) cells/mm^3^. Regarding viremia, the global median was 5.0 (3.5–5.3) log10 copies/mL, which was common throughout the three treatment lines, with varying interquartile ranges (IQR) (5 (4.2–5.5) log10 copies/mL, 5 (3.8–5.3) log10 copies/mL, and 5 (4.3–5.6) log10 copies/mL). According to the ART regimen, the majority of patients on the first line received TDF + 3TC + EFV (63.2%), followed by AZT + 3TC + NVP (31.3%); while for those on the second line, the majority received either the LPV/r or ATV/r-containing regimen (66.8% and 29.8%, respectively). Among those on the third line, the majority took DTG (75.9%) (Table 1). Regarding the level of adherence to ART at the time of genotypic resistance testing, the rate of good adherence was 9.1% (106/1170), poor adherence was 52.7% (617/1170), and people with no information on adherence had a rate of 38.2% (447/1170). Overall, the participants had a median duration of infection of 124 (76–180) months, the duration of treatment was 36 (14–72) months, and, regarding the clinical stage according to the CDC’s categorization, 14.4% (168/1170) were asymptomatic, 8.0% (94/1170) were moderate, 13.8% (162/1170) were advanced, 7.7% (90/1170) were severe, and, finally, 56.1% (656) had no information on their clinical stage.

### 3.2. Viral Subtype Distribution and Reverse Transcriptase Drug Resistance Mutations

**Figure 1:**
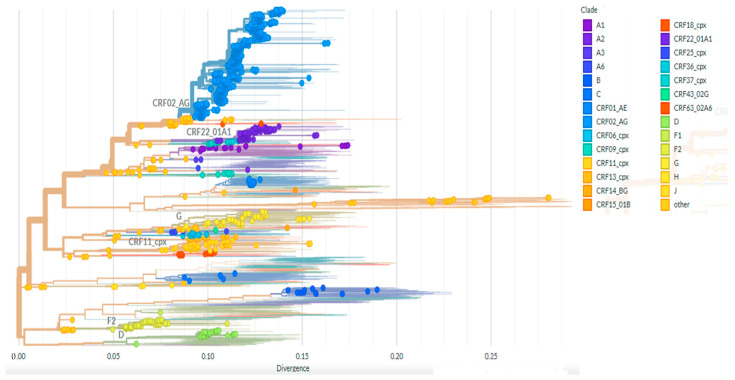
Phylogenetic tree of the 1170 viral subtype distribution among HIV-1 infected patients failing ART, inferred using Nextstrain software version 3.10.0 [26] (https://clades.nextstrain.org, accessed on 8 February 2024).

Among the 1170 study participants, a diverse distribution of HIV-1 non-B clades was observed, with CRF02_AG being the most prevalent (60.6%), followed by subtypes A1 (9.49%), G (5.81%), F2 (4.44%), and CRF11_cpx (2.14%). Additional combinations included A1 + G (2.48%), A1 + J (1.54%), CRF18_cpx (1.54%), and CRF01_AE (1.03%). Additionally, eleven participants (0.94%) were found to carry the B subtype (Figure 1).

**Figure 2:**
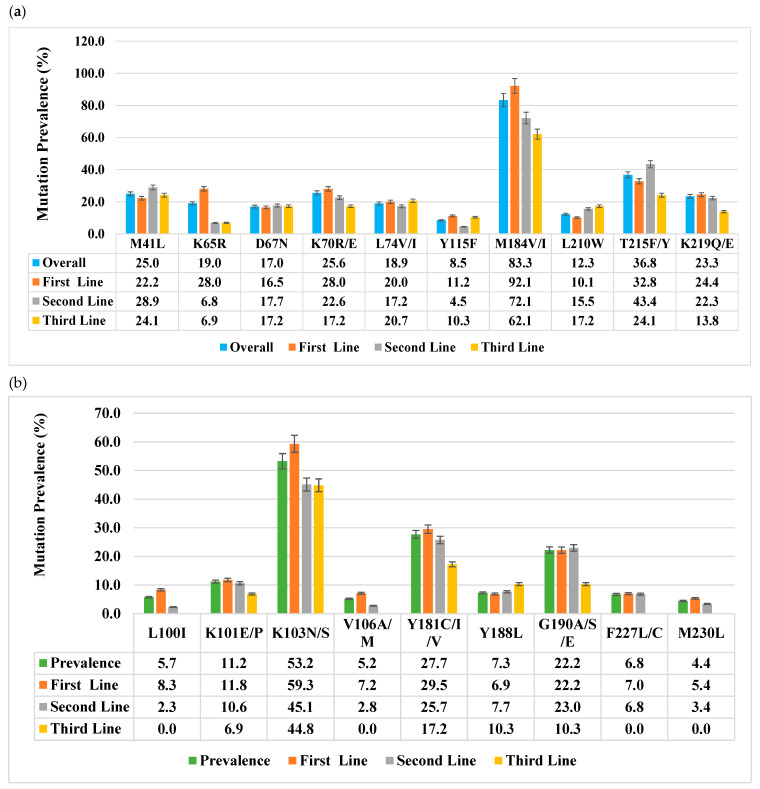
(**a**) NRTI and (**b**) NNRTI major drug resistance mutations’ prevalence through the treatment lines, with the overall prevalence in grey.

By analyzing the reverse transcriptase portion among patients failing first-, second-, and third-line ART, we identified 15 NRTI and 17 NNRTI major drug resistance mutations (DRMs) (Figure 2). The overall prevalence of reverse transcriptase drug resistance mutation was 77.4% for NRTI DRMs and 49.2% for NNRTI DRMs. NRTI DRM prevalence decreased across treatment lines, with 83.6%, 69.6%, and 62.1% observed in first-, second-, and third-line patients, respectively *p* < 0.001. Similarly, NNRTI DRM prevalence decreased across treatment lines, with 56.3%, 39.4%, and 41.4% observed in first-, second-, and third-line patients, respectively. The most frequent NRTI DRMs were M184V/I (83.3%), which was most prevalent among first-line patients; M41L (25.0%), which was highest among second-line patients; and T215F/Y (36.8%), which was most prevalent among second-line patients as well. For NNRTIs, the most frequent DRMs were K103N/S (53.2%), which was most prevalent among first-line patients; Y181C/I/V (27.7%), which was also highest among first-line patients; and G190A/S/E (22.2%), which was most prevalent among second-line patients. Overall, both NRTI and NNRTI mutations showed decreasing rates from the first to the third lines through the second line (Figure 2).

### 3.3. Antiretroviral Drugs Overall Susceptibility Rate

**Figure 3:**
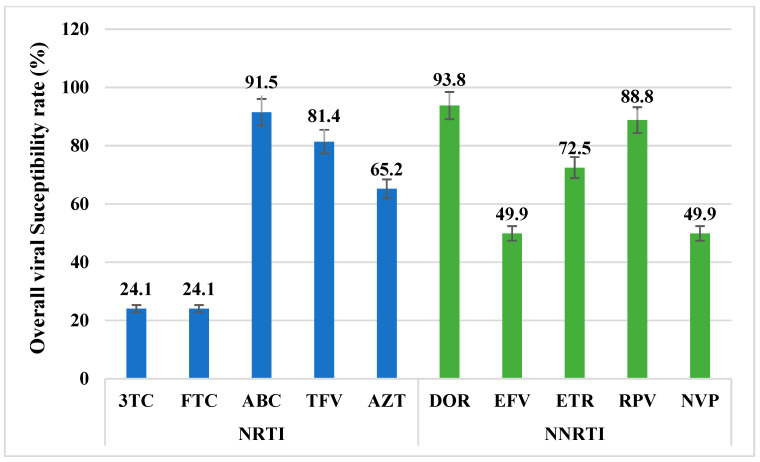
Predictive antiretroviral susceptibility in Cameroon, derived from mutation prevalences. blue NRTIs; green NNRTIs.

Based on the mutation profile and their prevalence in our study population, we assessed the overall susceptibility of each antiretroviral agent according to the Stanford list of mutations [29]. For NRTIs, the highest overall susceptibility was observed for ABC (91.5%), followed by TDF (81.4%) and AZT (65.2%). In contrast, 3TC and FTC both exhibited a susceptibility of 24.1%. For NNRTIs, the leading active agents were DOR (93.7%), RPV (88.8%), and ETR (72.5%), while EFV and NVP both had a susceptibility of 49.9% (Figure 3).

### 3.4. Covariation of Major Reverse Transcriptase DRMs with M184V Mutation

Another goal of our study was to assess the covariation of M184V mutations with other major NRTI and NNRTI DRMs observed in the reverse transcriptase gene among 1170 RTI-treated patients, according to the Stanford list of mutations [29]. To identify significant patterns of pairwise correlations between M184V and other major RT mutations observed in isolates from RT-treated patients, we calculated the binomial correlation coefficient (phi) and its statistical significance for each pair of mutations (Table 2).

### 3.5. Major Reverse Transcriptase DRMs Involved in Positive Correlations with M184V

Among first-line-failing patients, eight major RTI DRMs showed significant (adjusted *p* < 0.001) positive correlations with M184V mutation. In particular, NRTI DRMs were K70R (phi = 0.19, *p* < 0.001), L74I (phi = 0.18, *p* < 0.001), M41L (phi = 0.19, *p* < 0.001), T215Y (phi = 0.18, *p* < 0.001), T215F (phi = 0.18, *p* < 0.001), and K219E (phi = 0.14, *p* < 0.008), while NNRTIs were K103N (phi = 0.16, *p* < 0.004) and G190A (phi = 0.12, *p* < 0.05). Among second line-failing patients, M184V showed significant positive correlations with eleven mutations: T215Y (phi = 0.34, *p* < 0.001), M41L (phi = 0.33, *p* < 0.001), D67N (phi = 0.28, *p* < 0.001), T215F (phi = 0.28, *p* < 0.001), L210W (phi = 0.26, *p* < 0.001), K70R (phi = 0.26, *p* < 0.001), L74I (phi = 0.20, *p* < 0.001), K219E (phi = 0,19, *p* < 0.001), and K219Q (phi = 0.19, *p* < 0.001) for NRTIs and G190A (phi = 0.22, *p* < 0.001), K103N (phi = 0.16, *p* < 0.05), and K103S (phi = 0.14, *p* < 0.05) for NNRTIs. Among all 29 participants in the third line, no mutation showed a significant correlation with M184V (Table 2).

### 3.6. Major Reverse Transcriptase DRMs Involved in Negative Correlations with M184V

Two mutations showed negative correlations in the first line: K65R (phi = −0.26, *p* < 0.001) for NRTIs and Y181I (phi = −0.16, *p* < 0.05) for NNRTIs. We observed no negative correlations in the second and third lines (Table 2).

### 3.7. Clusters of Correlated Mutations

Because pairwise analysis suggested that M184V is significantly correlated with many major RTI resistance mutations, we performed average linkage hierarchical agglomerative cluster analysis [30] to visualize this hypothesis more precisely.

**Figure 4:**
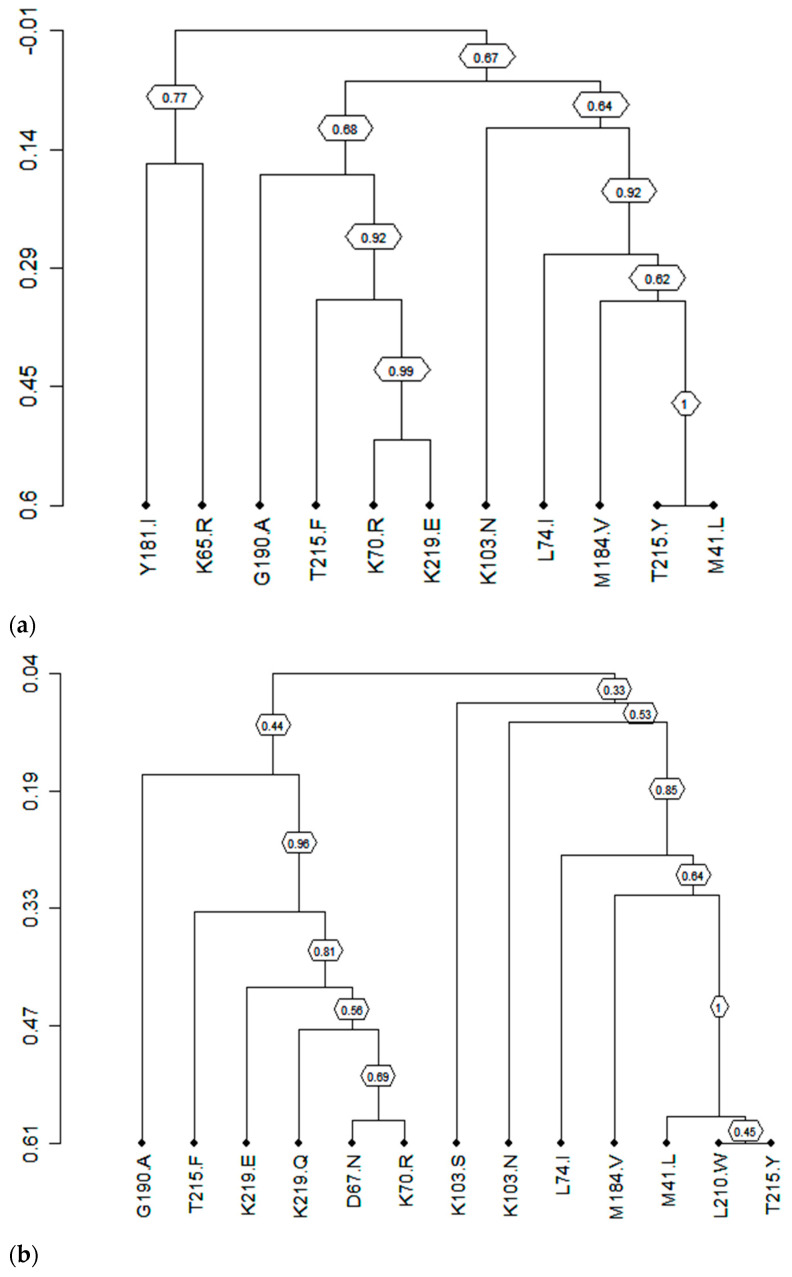

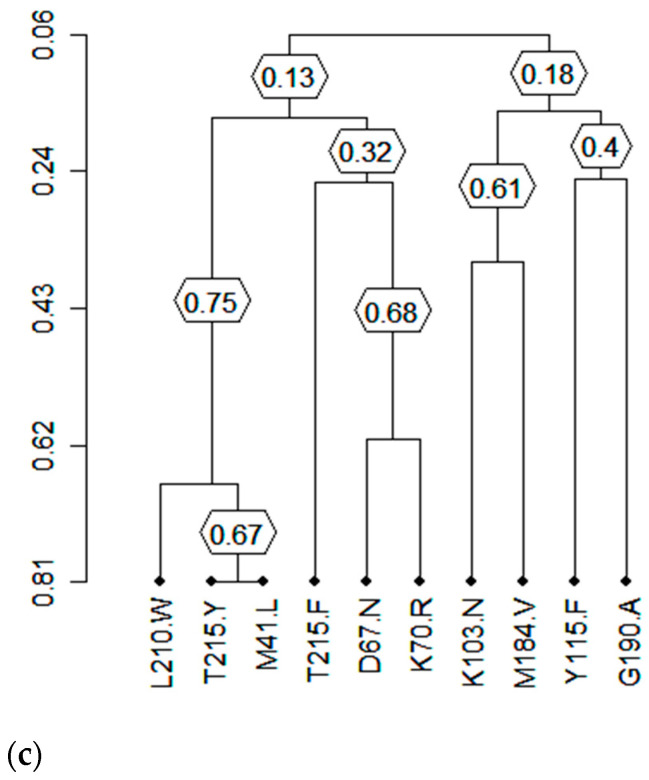
(**a**) Clusters of correlated mutations in first-line ART. (**b**) Clusters of correlated mutations in second-line ART. (**c**) Clusters of correlated mutations in third-line ART. Dendrogram obtained from average linkage hierarchical agglomerative clustering, showing clusters of RT mutations. The length of branches reflects distances between mutations in the original distance matrix. Bootstrap values, indicating the significance of clusters (≥0.2), are reported in the boxes.

In the first line, the dendrogram (Figure 4a) shows that M184V was significantly clustered (bootstrap value = 0.62) with T215Y and M41L (covariation frequency: 13.7% and 17.9%, respectively) (Table 2), and in the second line, it was significantly clustered (bootstrap value = 0.64) (Figure 4b) with M41L, L210, and T215Y (covariation frequency: 24.26%, 13.4%, and 20.0%, respectively) (Table 2). Among patients on the third line, M184V was significantly clustered (bootstrap value = 0.53) (Figure 4c) with K103N (covariation frequency: 28.9%) (Table 2).

## 4. Discussion

NRTIs have played a crucial role in HAART [31,32,33]. However, their widespread use has led to a rise in NRTI resistance [32]. Our findings highlight the substantial burden of drug resistance among treatment-experienced patients in Cameroon, with high prevalence rates for both NRTIs and NNRTIs, an overall prevalence of 77.4% and 49.2%, respectively. The high rates of resistance observed, particularly the dominance of the M184V/I mutation, which was present in 83.3% of NRTI-resistant cases, similar to the 83.2% observed by Takou et al., 2019 [17], suggest that previous treatment regimens have profoundly impacted the viral landscape, notably lamivudine and emtricitabine, which are highly affected by this mutation [34]. Therefore, given this high resistance, closely monitoring the tendency and co-occurrence between M184V and major reverse transcriptase mutations is a strong point for the development and strengthening of new treatment strategies such as combination therapies to combat evolving HIV drug resistance, especially in low- and middle-income countries (LMICs) like Cameroon, given the current treatment landscape, which is rapidly changing with the introduction of new antiretrovirals like Islatravir (ISL) [2,13,35,36].

This study provides valuable insights into the potential efficacy of ISL in combination with other ARVs within this context. Our analysis indicates that while ISL shows promise as a novel antiretroviral, the prevalence of the M184V/I mutation, which reduces ISL’s in vitro activity, is concerning [15,16,37]. This emphasizes the importance of considering this mutation in designing future treatment strategies. Our results revealed significant positive covariation between M184V and other major NRTI and NNRTI DRMs, especially in first- and second-line ART, suggesting that long-term exposure to specific antiretroviral classes, particularly NRTIs, might contribute to the emergence of M184V and potentially reduce the efficacy of ISL. Specifically, the positive correlation of M184V with several NRTI DRMs, among which a cluster is seen with L74I, T215F, and M41L in the first line and second lines, and additionally with T215Y and L210W in second-line ART, strongly suggests highly reduced susceptibility to AZT and its disqualification in combination with ISL [16,29,37]. Abacavir, despite keeping the second most important susceptibility rate (91.5%) in our study population, remains hugely affected by L74I, which happened to show significant positive correlations with M184V.

Concerning NNRTI DRMS, positive correlations were equally seen in clusters with K103N in the first line and K103S in the second line. This correlation eliminates EFV and NVP as treatment options in combination with ISL [16,29,37]. Doravirine, on the other hand, had the highest susceptibility rate (93.7%) in our study population; though Y188L, which is known to reduce viral susceptibility to DOR, was present (7.26%), it showed no significant correlation with M184V throughout all three treatment lines. This renders DOR the best potential NNRTI in combination with ISL [29,38,39,40].

On the other hand, M184V showed a negative correlation with K65R (NRTI) and Y181I (NNRTI) among first-line ART patients. This indicates the antagonistic relationship between M184V and K65R, showing the potential of K65R to enhance the activity of ISL [16,37,41] while reducing susceptibility to TDF. M184V, on the other hand, enhances susceptibility to TDF while reducing the susceptibility to 3TC/FTC and ISL [15,16,37], and the combined effect of M184V and K65R maintains the optimal activities of both drugs if administered together [15,16,37]. Despite Y181I showing antagonism with M184V in our analysis, it is known to reduce the efficacy of ETR, RPV, and NVP.

There were no significant correlations among patients failing the third line, which could be due to the low population size (29/1170). The dendrogram among these patients shows an M184V cluster similar to those of the first and second lines with K103N and G190A, further supporting the elimination of EFV and NVP in combination with ISL [16,29,37]. These results suggest that, given the resistance profile of our patients on treatment, the efficacy of Islatravir could be rapidly compromised if it is not combined with other molecules that retain good efficacy, and it would be an alternative treatment for the reverse transcriptase inhibitor class.

This study demonstrates that these co-occurrences not only challenge the management of HIV-1 but also highlight potential challenges for future treatment success. Though this approach has the potential to improve treatment outcomes and address the evolving landscape of HIV drug resistance in LMICs facing similar challenges, this study was essentially a predictive study and, therefore, requires further research to validate these findings in large cohorts and assess the clinical efficacy of these proposed combinations in Cameroon.

## 5. Conclusions

Our analysis of 1170 HIV-1 sequences from treatment-experienced patients in Cameroon revealed significant covariation patterns between the M184V mutation and key NRTI and NNRTI drug resistance mutations. These highlight the complex interplay of drug resistance development and underscore the urgent need for novel treatment strategies in low- and middle-income countries (LMICs) like Cameroon. Islatravir (ISL) holds promise as a potential therapeutic weapon, particularly when combined with tenofovir disoproxil fumarate (TDF) or Doravirine. Notably, Doravirine, which retains full activity against M184V, emerges as a particularly promising partner for ISL, offering a potential solution for patients failing ART who exhibit M184V and other resistance mutations in LMICs sharing similar programmatic challenges like Cameroon.

## Figures and Tables

**Table 1:** Socio–demographic characteristics; immuno–virological and treatment exposure of study population.

		First Line (*n* = 671)	Second Line (*n* = 470)	Third Line (*n* = 29)	Overall (*n*= 1170)	*p*-Value ^a^
**Gender**	Female (%)	437 (65.1)	264 (56.2)	13 (44.8)	714 (61.0)	0.032
	Male (%)	234 (34.9)	206 (43.8)	16 (55.2)	456 (39.0)	
**Age (years), median (IQR)**		41 [33–47]	39 [20–48]	17 [9–38]	40 [28–47]	<0.001
**CD4 (cells/µL), median (IQR)**		186 [63–361]	195 [58–374]	157 [63–665]	190 [63–665]	0.941
**Viral load (Log10 copies/mL), median (IQR)**		5 [4.2–5.5]	5 [3.8–5.3]	5 [3.4–5.3]	5 [3.5–5.3]	<0.001
**ART regimen *n* (%)**	2NRTIs + EFV	424 (63.2)	1 (0.2)	-	425 (36.3)	
	2NRTIs + NVP	210 (31.3)	-	-	210 (17.9)	
	2NRTIs + ATV/r	2 (0.3)	314 (66.8)	-	316 (27.0)	
	2NRTIs + LPV/r	-	140 (29.8)	-	140 (12.0)	
	2NRTIs + DRV/r	-	3 (0.6)	-	3 (0.3)	
	2NRTIs + NFV/r	-	1 (0.2)	-	1 (0.9 × 10^−1^)	
	2NRTIs + DTG	34 (2.9)	-	22 (75.9)	56 (4.8)	
	Others	1 (0.1)	11 (2.3)	7 (24.1)	19 (1.6)	

Note: IQR: Interquartile range; NRTIs: Nucleoside reverse transcriptase inhibitors; EFV: Efavirenz; NVP: Nevirapine; LPV/r: Lopinavir; ATV/r: Atazanavir; DRV/r: Darunavir; NFV/r: Nelfinavir; DTG: Dolutegravir. ^a^ Chi-squared independence test was used to estimate the potential differences.

**Table 2:** Significantly correlated pairs of M184V with HIV-1 RT major or accessory resistance mutations. The third line treatment shows a similar profile but there was no significance.

ART Line	RT Mutation	Frequency (%) ^a^	Covariated Mutations	Frequency (%) ^b^	Covariated Frequency (%)	phi	*p*-Value ^c^
I	M184V	561 (83.61)	K70R	122 (18.18)	84 (12.52)	0.19	6.69 × 10^−6^
			L74I	111 (16.54)	74 (11.03)	0.18	6.69 × 10^−6^
			T215F	109 (16.24)	88 (13.11)	0.18	1.89 × 10^−5^
			M41L	149 (22.21)	120 (17.88)	0.19	2.60 × 10^−5^
			T215Y	111 (16.54	92 (13.71)	0.18	4.79 × 10^−5^
			K219E	97 (14.46)	68 10.13)	0.14	7.38 × 10^−3^
			K65R	188 (28.02)	108 (16.10)	−0.26	2.15 × 10^−7^
			K103N	378 (56.33)	304 (45.31)	0.16	3.37 × 10^−3^
			G190A	135 (20.12)	106 (15.80)	0.12	4.91 × 10^−2^
			Y181I	9 (1.34)	1 (0.15)	−0.16	3.09 × 10^−2^
II		327 (69.57)	T215Y	110 (23.40)	94 (20.00)	0.34	9.73 × 10^−14^
			M41L	136 (28.93)	114 (24.26)	0.33	1.08 × 10^−11^
			D67N	83 (17.66)	75 (15.96)	0.28	1.73 × 10^−9^
			T215F	94 (20.00)	87 (18.51)	0.28	2.06 × 10^−9^
			L210W	73 (15.53)	63 (13.40)	0.26	4.93 × 10^−8^
			K70R	93 (19.79)	81 (17.23)	0.26	2.01 × 10^−7^
			L74I	61 (12.98)	50 (10.64)	0.20	2.35 × 10^−4^
			K219E	53 (11.28)	45 (9.57)	0.19	3.31 × 10^−4^
			K219Q	52 (11.06)	43 (9.15)	0.19	5.25 × 10^−4^
			G190A	97 (20.64)	84 (17.87)	0.22	3.49 × 10^−5^
			K103N	185 (39.36)	136 (28.94)	0.16	1.59 × 10^−2^
			K103S	27 (5.74)	21 (4.47)	0.14	2.77 × 10^−2^
III		18 (62.1)	T215Y	5 (17.24)	4 (13.79)	0.36	0.13
			D67N	5 (17.24)	2 (6.90)	0.36	0.13
			M41L	7 (24.14)	3 (10.34)	0.27	0.20
			K70R	4 (13.79)	3 (10.34)	0.31	0.27
			Y115F	3 (10.34)	3 (10.34)	0.27	0.27
			L210W	3 (10.34)	2 (6.90)	0.27	0.27
			T215F	2 (6.90)	1 (3.45)	0.21	0.27
			K103N	12 (41.38)	10 (34.48)	0.37	0.06
			Y188L	3 (10.34)	2 (6.90)	0.27	0.27
			G190A	3 (10.34)	4 (13.79)	0.27	0.27

Note: ^a^ The frequency was determined in 671, 470, and 29 isolates from first-, second-, and third-line ART, respectively. ^b^ Percentages were calculated for patients containing each specific mutation. ^c^ All *p*-values for covariation were significant at a false discovery rate of 0.05.

## Data Availability

Data are available on the CIRCB-CARE database, and can be shared upon request to interested researchers. Correspondence and requests for materials should be addressed to nkalexdurand@yahoo.com.
